# The role of microalgae culture modes in aquaculture: a brief opinion

**DOI:** 10.3389/fbioe.2023.1196948

**Published:** 2023-05-23

**Authors:** Pengfei Cheng, Shengzhou Shan, Zhujun Zhu, Kui Liu, Zorigto Namsaraev, Ivan Dubovskiy, Qingshan Xu

**Affiliations:** ^1^ Marine Drugs and Biological Products Department, Ningbo Institute of Oceanography, Ningbo, Zhejiang, China; ^2^ College of Food and Pharmaceutical Sciences, Ningbo University, Ningbo, China; ^3^ Kurchatov Centre for Genome Research, NRC “Kurchatov Institute”, Moscow, Russia; ^4^ Laboratory of Biological Plant Protection and Biotechnology, Novosibirsk State Agrarian University, Novosibirsk, Russia; ^5^ Lijiang Cheng Hai Bao Er Biological Development Co., Ltd., Lijiang, China

**Keywords:** microalgae, culture mode, aquaculture, mixotrophy culture, microalga culture strategies

## 1 Introduction

The aquaculture production in Asia accounted for approximately 92% of the global production, and China’s total aquaculture production accounted for >60% of Asian (Wang et al., 2020). The wide range of aquaculture contribute greatly to world food security and human nutrition (Britsch et al., 2021; Zhang et al., 2021). Bait cost accounts for a large proportion, which is an important factor to develop intensive economic breeding (Dourou et al., 2020). At present, the overall development level of bait is relatively simple, and some of them even need to be improved or imported (Apandi et al., 2019). Owing to the low quality and high cost, incomplete nutrition, and weak pertinence, many aquaculture producers choose to use inappropriate aquaculture bait, thereby leading to a series of resource and environmental problems (Hemaiswarya et al., 2011). Thus, aquatic bait has become a key factor restricting the development of the aquaculture industry.

Microalgae are a kind of plankton with wide distribution, rich nutrition, and high photosynthesis utilization (Sidari and Tofalo, 2019). The rich and balanced nutrients and various bioactive substances in microalgae can meet the nutritional requirements for the development of aquaculture animals at the seedling stage (Sicuro, 2021). Many studies have proven that rational use of bait microalgae has the comprehensive effects of improving survival rate, ensuring seedling development, improving growth rate, body length, weight, and immunity in the rearing of shrimp, shellfish, and sea cucumber (Vu et al., 2018). Microalgae have another important function of feeding secondary feed, such as for rotifers, halogenates, and copepods. They can significantly enhance the contents of PUFA and various vitamins in secondary feed organisms to meet the requirements of aquatic larvae (Sandeep et al., 2019). Also, microalgae play an important role, which is primarily reflected by the water quality and algal–bacteria phase quality (Salam et al., 2016). Microalgae can use photosynthesis to produce oxygen and absorb CO_2_, nitrogen, and phosphorus emitted by seedlings to control the “CO_2_-HCO_3_^-^” balance in water and stabilize pH (Esteves et al., 2022). Microalgae have the potential to play a significant role in aquaculture due to their high-quality protein, essential fatty acids, pigments, and other nutrients (Francis et al., 2001). When used as bait for aquatic organism, microalgae offer a superior amino acid content compared to fish meal and other animal feed (Seong et al., 2021). Additionally, microalgae can serve as a means of purifying aquaculture water and regulating microbial balance in the water. Therefore, microalgae can be utilized not only as a feed source for aquatic animals, but also as a tool to enhance the overall health and sustainability of aquaculture operations.

Currently, microalgae are commonly used as aquatic bait in more than 20 genera and 40 species, including golden algae, diatoms, green algae, and etc., (Sandeep et al., 2019). However, how to reduce the cost of algal culture and increase its density has become a problem puzzling for many years. In the process of culturing microalgae as biological bait, the main challenges are the high cost of cultivation, low harvest rate, and low biomass yield, which hinders the industrialization development of bait microalgae (Bo et al., 2023). However, it is possible to achieve the growth of low-cost and high-density microalgae with different culture mode. The main ways of microalgae culture are photoautotrophic, heterotrophic, and mixotrophic culture. In photoautotrophic mode, microalgae use chlorophyll or phycocyanin to convert light energy into the energy required by the Calvin cycle, or microalgae themselves provide substrates for the Calvin cycle to maintain growth (Hwang et al., 2014). In heterotrophy mode, microalgae use organic carbon and nutrients through their own Calvin cycle for aerobic respiration to obtain energy for cell metabolism (Barros et al., 2019). Under mixotrophy culture, microalgae can use of organic and inorganic carbon to keep their metabolism at a higher level (Zhang et al., 2021). Currently, microalgae are primarily cultivated using photoautotrophic mode due to its low energy consumption. However, this method often results in limited biomass accumulation due to inadequate light exposure. Although heterotrophic culture may have a higher biomass than photoautotrophic culture, it is faced with several challenges, including the high cost of organic carbon, long growth cycle, and the harmful bacteria. Mixotrophic culture, which combines both photoautotrophic and heterotrophic cultures, is currently being extensively researched, but it has not yet been implemented on a large scale. Therefore, selecting a suitable culture mode for microalgal growth is important, but also need to be provided an in-depth discussion of the application of microalgae in aquaculture (Sicuro, 2021).

## 2 Application of different microalga cultivation modes in aquaculture

### 2.1 Microalga photoautotrophy culture mode

For photosynthesis, microalgae fixed CO_2_ through light absorption, electron transport, photosynthetic phosphorylation, and carbon assimilation. Then, they convert light energy into usable reduced coenzyme II [NAD(P)H] and ATP (Sun et al., 2016). CO_2_ and light (light period and light intensity) are the main factors affecting the growth of photoautotrophic microalgae, and light energy is converted into cellular substances (Butti and Mohan, 2018). In aquaculture, photoautotrophic microalgae have obvious economic advantages. There is no addition of carbonate, which will not lead to excessive alkaline problems in the water (Hwang et al., 2014). However, the photoautotrophic mode for some species of microalgae has obvious disadvantages in aquaculture. It depends substantially on external conditions, and when the external solar energy is low, the time is shortened or a large interval exists (Hwang et al., 2014). The shortage of solar energy can also reduce microalga density and make it easier for bacteria to multiply (Yu and Kim, 2017). In photoautotrophy culture, most algal cells catabolize self-produced nutrients such as polysaccharides, lipids, and proteins to survive, resulting in lower algal biomass than other culture modes (Simal-Gandara et al., 2022).

### 2.2 Microalga heterotrophy culture mode

For heterotrophy culture, microalgae absorb external organic carbon to synthesize biomass and reproduce under dark conditions (Pribyl and Cepak, 2019). Heterotrophs do not rely on external inorganic carbon sources and light energy. Thus, photosynthesis is reduced, but cell density and biomass relatively increase (Fan et al., 2012). However, not all microalgae are capable of heterotrophic growth. The main reason is that they do not have perfect mechanisms for the uptake and utilization of extracellular organic carbon and organic nitrogen (Nzayisenga et al., 2018). Specifically, some microalgae cannot be heterotrophic owing to the difficulty in entering the cell or lack of an ability to concentrate organic matter (Di Caprio et al., 2018). Moreover, the enzyme system required for the metabolism of organic matter in the cell is not perfect, and organic matter cannot be effectively used, which makes it difficult for some microalgae to heterotrophy (Han et al., 2012). Also, some microalgae cannot heterotrophy owing to insufficient energy provided by respiration to sustain their growth (Zhou et al., 2017). Microalga heterotrophy mode has great advantages in aquaculture. They can also rely on organic carbon sources to provide energy through the tricarboxylic acid cycle to reproduce, thereby avoiding the reproduction of bacteria by using nutrients in deep water (Charoonnart et al., 2018). On the other hand, microalga biomass is always larger than that under photoautotrophic conditions, which may be due to the exogenous addition of organic carbon to preserve the nutrients produced (Toh et al., 2012). However, the heterotrophy mode also has some disadvantages. For example, owing to the organic carbon, bacteria and fungi easily use reproduction to antagonize microalgae. Also, different microalgae adapt to different types of organic carbon, but the expensive glucose remains the main carbon source (Azra et al., 2022).

### 2.3 Mixotrophy culture mode of microalgae

**FIGURE 1:**
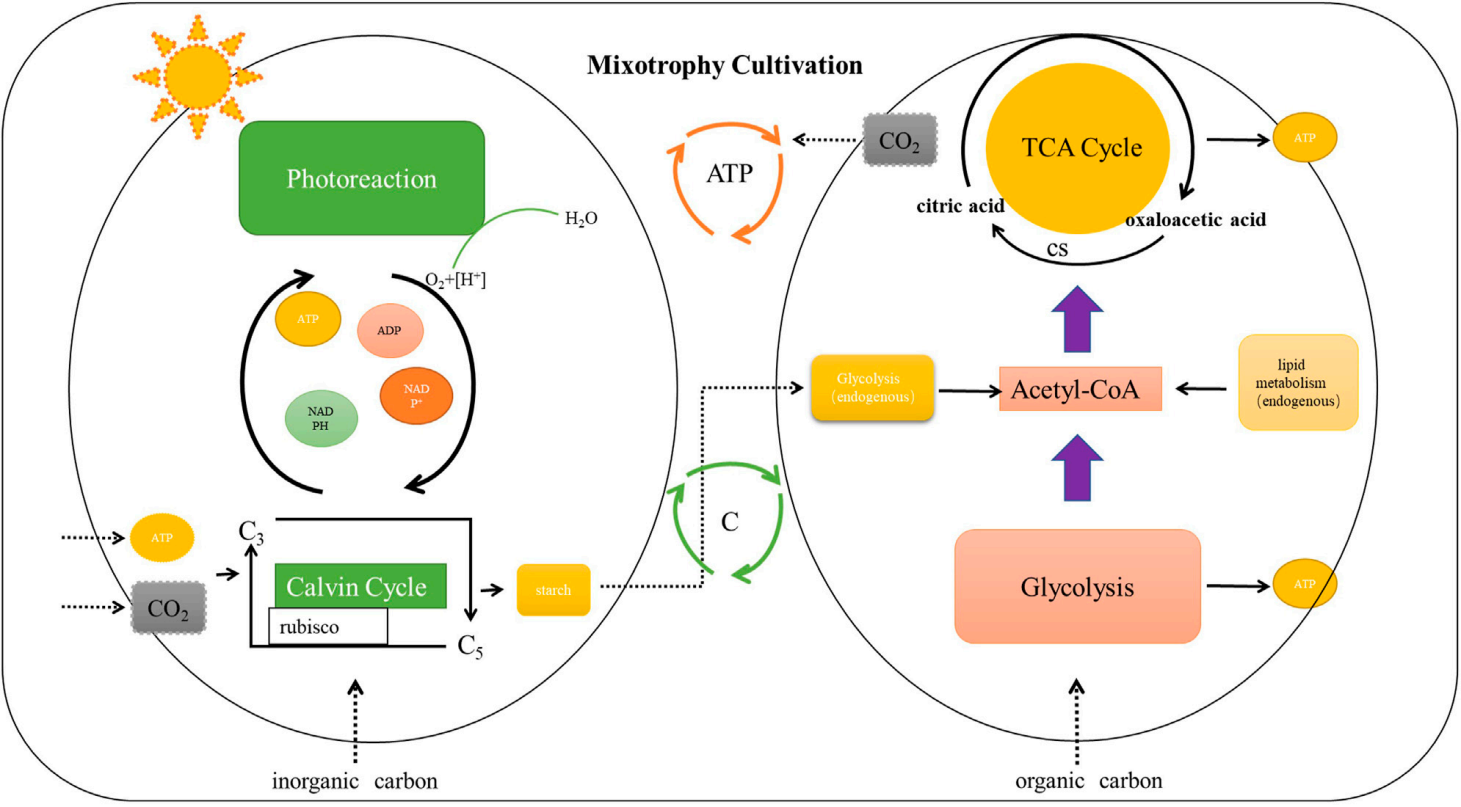
The Mechanism diagram for mixotrophy culture of microalgae.

For mixotrophy culture, microalgae can use light energy and external inorganic carbon to provide required CO_2_ for photosynthesis and also absorb external organic carbon to provide energy for growth (Figure 1). Mixotrophy culture is a complementary mode of photoautotrophy and heterotrophy (Zhang et al., 2021). In this mode, microalgae can obtain the energy required for the dark reaction and the oxygen required for the tricarboxylic acid cycle and can use the inorganic carbon to store the dark reaction products for catabolism (Zhan et al., 2017). Compared with other two modes, mixotrophy culture has obvious advantages. Firstly, most microalgal mixotrophy cultures have higher density and biomass than photoautotrophy and heterotrophy modes (Zanette et al., 2019). Moreover, active substances that are unavailable in the photoautotrophy and heteromorphy are often obtained in this mode after being eaten by farmed animals, and the quality significantly improves (Wang et al., 2016). However, the conditions of mixotrophy culture mode are strict, such as light source, inorganic carbon, organic carbon, and temperature suitable for microalga growth (Verma et al., 2020). Also, the cost requirement of mixotrophy mode is always higher than that of other modes (Roostaei et al., 2018). Some studies have found that the consumption of inorganic carbon in mixotrophy culture is slightly lower than that in photoautotrophy culture, and the consumption of organic carbon in mixotrophy culture is slightly lower than that in heterotrophy culture, which may be due to the enhanced light-energy utilization (Liaqat et al., 2022). Mixotrophy culture is a better mode for most microalgae, however; it may not be completely suitable for aquaculture owing to harsh conditions and high cost. For the mixotrophy culture, equipment modification and technical limitations may lead to increased production costs, and whether it can generate benefits needs further exploration. At present, the mixotrophy culture of microalgae in aquacultural water should avoid biological pollution such as miscellaneous bacteria in water. On the basis of avoiding biological destruction, screening beneficial microorganisms that promote the cooperative growth of microalgae, constructing efficient mixed culture system and increasing its commercial value are also urgent solutions.

## 3 Conclusion

The different microalga-cultivation modes in aquaculture activities still face several urgent problems that need to be solved. Photoautotrophic mode is low cost but has the low biomass. Under the premise of controllable environmental effects, the growth efficiency of microalgae can be improved by regulating the CO_2_-utilization rate. In heterotrophy, some microalgae have higher biomass, but the addition of organic carbon enables its easy use by bacteria. Heterotrophic microalgae can adapt to deep aquaculture waters, which are more conducive to high-density or large-scale aquaculture culture. However, the selection of heterotrophy microalgal strains and the cost of organic carbon sources are crucial issues. In mixotrophy culture, most microalgae have higher biomass and bioactive substances, and the uptake of organic carbon and inorganic carbon in the environment may be reduced compared with other two modes, which may save on costs from the point of view. Importantly, in mixotrophy, the light-reaction intensity of microalgae is significantly higher than that in the photoautotrophy and heterotrophy modes. Thus, the oxygen production of microalgae significantly increases, playing a role in killing anaerobic bacteria in water and forming a “complementary” condition with the CO_2_ produced by farmed animals. The biomass and nutrient accumulation of microalgae varied depending on the different culture method. For instance, mixotrophy culture conditions can promote the accumulation of polyunsaturated fatty acids and other nutrients, which can serve as high-quality aquatic food and ensure the successful development of aquatic animal’s larvae.

In summary, selecting the appropriate culture mode for microalgae in aquaculture is important. It should be considered in terms of their own reproduction and the water body or animals being farmed. Furthermore, specific problems should be specific analyzed such as breeding season, temperature, and light to achieve mutual-symbiosis conditions between multiple species and microalgae in the breeding area.
